# Mechanical and Tribological Properties of Aluminum-Based Metal-Matrix Composites

**DOI:** 10.3390/ma15176111

**Published:** 2022-09-02

**Authors:** Avinash Lakshmikanthan, Santosh Angadi, Vinayak Malik, Kuldeep K. Saxena, Chandar Prakash, Saurav Dixit, Kahtan A. Mohammed

**Affiliations:** 1 Department of Mechanical Engineering, Nitte Meenakshi Institute of Technology, Bangalore 560064, India; 2 Department of Mechanical Engineering, KLS Gogte Institute of Technology, Belagavi 590008, India; 3 Department of Mechanical Engineering, GLA University, Mathura 281406, India; 4 School of Mechanical Engineering, Lovely Professional University, Phagwara 144001, India; 5 Division of Research and Development, Lovely Professional University, Phagwara 144001, India; 6 Peter the Great St. Petersburg Polytechnic University, 195251 Saint Petersburg, Russia; 7 Division of Research & Innovation, Uttaranchal University, Dehradun 248007, India; 8 Department of Medical Physics, Hilla University College, Babylon 51002, Iraq

**Keywords:** Al-based MMCs, mechanical, tribological, dual, particle, reinforcement, applications

## Abstract

This review article focuses on the aluminum-based metal matrix composites (Al-based MMCs). Studies or investigations of their mechanical and tribological properties performed by researchers worldwide in the past are presented in detail. The processing techniques and applications for Al-based MMCs are also documented here. A brief background on the composite materials, their constituents, and their classification, as well as the different matrix materials and particulates used in Al-based MMCs, can be found in this review. Then, an overview of dual-particle-size reinforced composites, heat treatment of Al alloys, and temper designations used in heat treatment are also included. In addition, the factors influencing the mechanical and wear properties of Al-based MMCs are discussed. The primary objective is that both present and future researchers and investigators will be assisted by the comprehensive knowledge compiled in this article to further explore and work towards the betterment of society in general.

## 1. Introduction

Composite materials are engineering materials made by a mixture of different constituents that are distinct from each other and offer several properties that are unattainable by their individual constituents [1,2]. In composite materials, the continuous phase is known as the matrix phase, while the reinforcing phase is generally discontinuous and can have different forms based on its aspect ratio. Based on the inputs of a design engineer, the composite material can be tailored to fit certain requirements by opting for appropriate materials [3].

**Figure 1:**
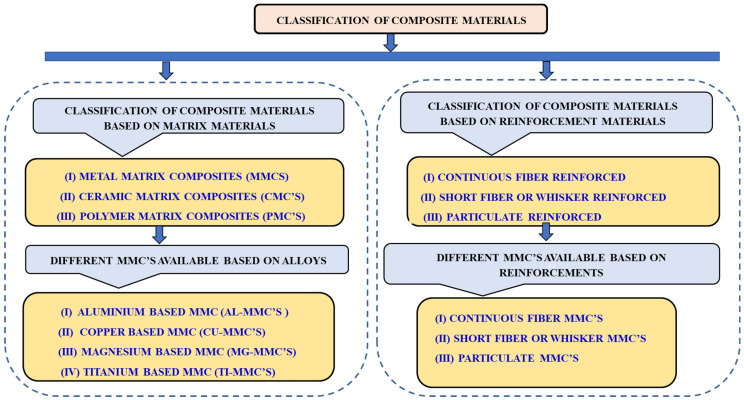
Flowchart showing the broad classification of composite materials.

Composite materials can be classified based on their matrix and reinforcement materials. Based on the morphology of the reinforcements, the composites can be classified as fiber, particulate, whisker, or flake. Based on the type of matrix, composite materials are classified into metal-matrix composites (MMCs), ceramic-matrix composites (CMCs) and polymer-matrix composites (PMCs) [4,5,6,7]. A broad classification of the composite materials is shown in Figure 1.

Based on the matrix material used, composite materials are classified as follows:

**Metal-Matrix Composites (MMCs):** Metal matrix composites consist of two main components. The first is metal/alloy matrix, which is the continuous phase, and the second is the reinforcement, which is the discontinuous phase and can have different morphologies. Commonly used metals and alloys are aluminum, nickel, copper, steel, titanium, and magnesium, which are used for numerous applications spanning from electrical wire to automobile parts. With respect to polymer-matrix composites (PMCs), MMCs offer high specific strength, high wear resistance, ability to deform plastically, better joining characteristics, and enhancement of strength by various methods.

**Ceramic-Matrix Composites (CMCs):** Non-metallic solids with strong ionic bonding are generally known as ceramics and are crystalline in nature. Ceramics possess high temperature operating capability, high chemical inertness, high compressive strength, and high hardness. However, due to their poor fracture toughness, they are not suitable for structural applications. To improve their fracture toughness, ceramics are reinforced with fibers (Al_2_O_3_, ZrO_2_, BN, Si_3_N_4_), whiskers (glass, TiB_2_, SiC, Si_3_N_4_), and particulates (Al_2_O_3_, TiC, SiC). Ceramic matrix composites are used for making cutting tool inserts, aircraft engines, brake disks, turbine blades, exhaust ducts, heat exchanger tubes, and bearings.

**Polymer-Matrix Composites (PMC’s):** Here, an organic polymer matrix is reinforced by different types of continuous or short fibers or sometimes even nanoparticles. Two different categories of polymer-matrix composites, namely reinforced plastics and advanced polymer composites, have been developed. These two polymer composites are distinguished on the basis of their achieved strength and stiffness. Polymers themselves are classified as thermoplastics (PEEK, polyetherimide, and liquid crystal) and thermosets (polyamides, epoxy, and polyester). On the other hand, reinforcements such as continuous fibers (aramid, glass, and carbon) and nanoparticles (TiO_2_, Al_2_O_3_ and CaSiO_3_) are used to reinforce polymers. Common applications include non-structural parts such as exterior panels, structural parts such as leaf spring for vehicles, molded propeller assemblies in the naval industry, lampposts, helicopter rotor blades, golf club shafts, and highway culverts for construction purposes.

### 1.1. Composite Materials Based on Reinforcement Material

Reinforcements are the materials used to strengthen the matrix phase by various strengthening mechanisms. These are used to reinforce the matrix because of their advantages, such as low density, thermal stability, high stiffness, high hardness, and high strength [8]. Generally, reinforcements are classified as continuous fibers, whiskers, or particulates based on their aspect ratio and morphology. Based on the application, reinforcements with similar property profiles are chosen to make a part/component. Fibers are quite popular owing to their high strength, high aspect ratio, and a high degree of flexibility. Various fibers such as glass fibers (E/C/S glass), boron fibers, aramid fibers, carbon fibers, SiC fibers, and α-alumina fibers are used as reinforcements [9]. Monofilament fibers have high elastic modulus, while carbide fibers possess both high specific modulus and strength. SiO_2_- and SiC-based multifilament fibers possess very high specific strength when compared to that of a specific modulus. On the other hand, whiskers are monocrystalline, which means they have no grain boundaries, due to which they possess very high strength and high stability and are not degradable. However, their main drawback is the lack of uniform dimensions. Another major drawback of whiskers is poor bonding with the matrix materials; for example, Al_2_O_3_ whiskers have difficulties in bonding with metal matrix. Among all available whiskers, silicon carbide and silicon nitride whiskers are quite popular for reinforcing metal matrices. However, one should note that whiskers are known to be hazardous to health, causing serious problems. Since MMCs with particulate reinforcements cost much less than MMCs with continuous fiber reinforcements, the particulate composites are more popular. From the processing point of view, the particulates can be blended more efficiently with the metal matrices when compared with that of whiskers or fibers. Ceramic-based particulate materials are quite popular owing to their low density, high strength, thermal stability, and good mechanical compatibility [10,11].

**Continuous-Fiber MMCs:** The reinforcing phase, in this case, is continuous fiber whose aspect ratio is more than 1000 and whose diameter can vary from 3 to 150 µm. Examples of continuous fiber MMCs are Al-SiC, Al-Al_2_O_3_, Al-B, Ti-6Al-4V-SiC, Al-Li-Al_2_O_3_, Mg-Al_2_O_3_, and Mg-C composites [12].

**Short-Fiber or Whisker MMCs:** The reinforcing phase is a short fiber or a whisker whose aspect ratio is usually over 10 and whose diameter can be in the range of 0.01 to 1 µm. Examples of short-fiber/whisker MMCs are AlSi_12_CuMgNi/Al_2_O_3_ short fibers, AlSi_12_CuMgNi/SiC whiskers, and Al/SiC whisker composites [12].

**Particulate MMCs:** Here, the reinforcing phase is a particle whose aspect ratio lies in the range of 1 to 4, with its size varying from nanometer to micrometer. Common examples of particulate MMCs are Mg/SiC, Pb/Al_2_O_3_, AlMgSiCu/Al_2_O_3_, Al-4Cu-2Mg/SiC, and Al-12Si-Mg/SiC composites [13,14,15]. Table 1 provides the information related to the types of reinforcements in terms of the aspect ratio. In addition, the existing examples for each type are also listed here.

### 1.2. Composites Based on Various Metals/Alloys Available as Matrix Materials

There are various types of metals or alloys available that can be used as matrix materials for making MMCs depending upon the applications. A brief discussion on different matrix materials used to develop MMCs is provided below [3,11,16].

**Aluminum and its Alloys:** Aluminum and its alloys are widely used because of their low density (2.7 g/cm^3^) and reasonable mechanical properties, such as good strength and corrosion resistance. The alloy series such as Al2xxx, A16xxx, and Al7xxx hold special significance when subjected to heat treatment. After heat treatment, these alloys exhibit good strength and toughness, which make them probable candidate materials for the automotive and aircraft industries.

**Copper and its Alloys:** Copper has a density of 8.9 g/cm^3^. This high density value rules out its application in automotive or aircraft industries as structural components. However, what are more important are its physical properties, such as electrical and thermal conductivity. Due to its high ductility, Cu can be easily cast and worked using secondary processing techniques. In its pure state, it is used for thermal management and electrical applications such as heat sinks, power cables, bus bars, and other electrical components.

**Magnesium and its Alloys:** This is one of the lightest metals, whose density (1.8 g/cm^3^) is less than that of aluminum. It possesses high specific strength, good damping properties, and good electrical conductivity. Furthermore, it can be cast or formed easily, due to which it is seen as an attractive material for large-volume applications.

**Titanium and its Alloys:** The density of titanium is 4.5 g/cm^3^, which is lower than iron. Ti exhibits a higher melting point than steel. Ti and its alloys are preferred for structural applications where the temperature ranges from 425 to 595 °C. This metal is passive to almost all mineral acids and chlorides, thereby providing a high degree of corrosion resistance. Microstructure and chemical composition are the two important factors that determine the properties of titanium alloys. Applications of titanium and its alloys range from compressor blades and armor vehicles to hip implants.

**Particulates used to Reinforce the Metal Matrices:** Hard particulate materials, such as borides, nitrides, oxides, and carbides, are used to reinforce the metal matrices. Table 2 shows the various reinforcements along with their crystal structure, density, melting point, and physical properties. It can be seen from Table 2 that these reinforcements have very low density but high melting point and elastic modulus. These economically produced ceramic reinforcements are currently being used in the polishing and grinding industries [13,14,15].

### 1.3. Importance of Metal Matrix Composites

Day by day, increasing concerns for the environment, the depletion of fossil fuels, and the need for advanced engineering materials have posed a big challenge to researchers across the world. Live examples from nature and continuous efforts have led to the development of composite materials. The basic components of a composite material are the matrix phase, which is continuous, and the reinforcement, which is discontinuous. Both phases are chemically distinct and capable of imparting a new range of properties that are otherwise not achieved by individual constituents of the composite material. This new class of materials can be distinguished based on the type of reinforcement or matrix material. The MMCs offer competitive advantages such as improved specific strength, specific stiffness, high toughness, high surface durability, improved joining characteristics, and thermal stability [17,18]. Initially, MMCs found applications in the aerospace industry, followed by their expansion into non-military fields such as the transportation industry [19]. For example, stainless-steel-fiber-reinforced aluminum composites were used for making connecting rods for automobiles [20,21]. Some companies have made tennis racket frames and golf clubs using silicon-carbide-reinforced aluminum composites. Apart from this, some special applications of MMCs include superconducting magnets made from copper-reinforced Nb-Ti filaments and oil-drilling inserts made up of tungsten-carbide-reinforced cobalt composites.

It is well known that the matrix material in MMCs is either a metal or alloy, while the reinforcement could be either ceramic or metal. Most commonly preferred metal matrices are based on aluminum, copper, cobalt, magnesium, nickel, silver, titanium, and zinc. Aluminum and magnesium are quite popular in the aerospace and automotive industries due to their low densities [20,21]. Due to their high thermal and electrical conductivity, copper and silver are used in thermal management applications and superconductors. On the other hand, the reinforcement is classified based on the aspect ratio and morphology such as particulates, whiskers, and short and continuous fibers [22]. However, it is most important to note that the ceramic-based reinforcements are quite popular for reinforcing MMCs because of their high service temperature, high strength, and elastic modulus. The reinforcements in the form of particulates are most preferred compared to continuous fibers as the cost of these high-modulus fibers is very high. On the other hand, particulates such as silicon carbide also have a modulus of about 410 GPa, along with a low density of 3.21 g/cm^3^. In addition to this, particulates are produced by very inexpensive techniques, such as crushing. Based on the application point of view, both matrix and reinforcement are chosen judiciously to meet the design engineer’s requirements for an application. Apart from the selection of constituents of a composite material, their fabrication also plays a prominent role in dictating the properties, as well as its affordability in the commercial market. There are various techniques available for fabrication, from conventional liquid metallurgy and powder metallurgy to newer techniques such as thermal spray and accumulative roll bonding [23]. However, the economical fabrication of composites is very crucial in realizing the advantages offered by them and to compete with other engineering materials. As of now, they are restricted to few applications, such as the automotive industries, where high strength-to-weight ratio is a major concern.

## 2. Aluminum Metal-Matrix Composites (AMMCs)

Al-based MMCs are one of the promising materials for applications in the automotive and aerospace industries as they exhibit low density, high specific strength, and good wear and corrosion resistance [24,25]. Aluminum alloys such as Al-Si, Al-Mg-Si, Al-Zn-Mg, Al-Cu, and Al-Cu-Mg have been used as matrix materials in the development of AMMCs. The addition of reinforcements (Al_2_O_3_, TiC, SiC, etc.) to aluminum matrix helps in deflecting the crack at the interface and thereby ceases it from propagating, resulting in an increase in its toughness. In addition, the reinforcement material may help in bridging the cracks, thereby improving the performance of the aluminum matrix. Out of all reinforcements, SiC particulate is quite popular because of its high-temperature stability, high strength, and stiffness [26,27].

### 2.1. Cast Aluminum Alloys

Cast aluminum alloys are strengthened by the same alloying elements and by the same mechanisms as wrought aluminum alloys [28]. Cast aluminum alloys are classified into heat-treatable and non-heat-treatable alloys with the same temper conditions. In general, aluminum alloys are identified by three- or four-number designation systems, with or without a decimal point in between them. Wrought and cast alloy systems are differentiated by the decimal point. There is a decimal point after three numbers in the designation of cast alloys, while there is no decimal point in the designation of the wrought alloy system. The numbering system is based on the alloying constituents and is preceded by letter “A”, which represents aluminum. The designation of cast aluminum alloys is shown in Table 3.

The 6xxx series of aluminum wrought alloys are used extensively, whereas the cast alloys of 6xx.x and 9xx.x series are rarely used. Cast alloys contain a large weight percentage of alloying elements, which results in a heterogeneous structure when compared to wrought alloys [29]. However, the addition of alloying elements to aluminum requires a detailed study as it can result in brittle phase formation. The brittle phase has a sharp morphology, which is capable of creating internal notches. These, in turn, facilitate crack nucleation under loading conditions. Proper selection of alloying elements and good processing conditions can prevent such defect formation. Since the present work employs cast aluminum alloys as matrix material, detailed information, from the structure to the physical properties, is provided in the upcoming sections.

### 2.2. Cast Aluminum Alloy A357

In order to make any component or part for an instrument or heavy machinery, a material with good castability or formability is often preferred. In case of cast aluminum alloys, the Al-Si-Mg alloys are well known for their excellent castability, due to which they are quite popular in the automotive industry. It is interesting to note that up to 85% of cast aluminum products are made up of Al-Si alloys. Hence, these alloys are extremely important in casting industries related to aluminum [30,31]. The Al-Si alloys are generally designated as the A3xx.x family. Among these, Al-Si hypoeutectic (<11%-Si) alloys such as A356 and A357 are quite popular. In particular, the structural components of automobiles, such as frames, housings, brackets, and car wheels, are made up of cast Al-Si alloys, such as A357. Further, A357 alloy is replacing cast-iron-based components such as cylinder liners due to its high wear resistance and physical characteristics [19]. A357 alloy is a cast aluminum alloy composed of 6–7.5% silicon, 0.4–0.7% magnesium, 0.04–0.07% beryllium, 0.12% iron, and 0.10% copper. Here, the addition of each element has its own significance: Si improves the fluidity, Mg is added as a precipitation hardener, Ni is added to increase tensile strength, copper increases the high-temperature properties, and Mn is added to modify Fe intermetallics. Its microstructure consists of α-Al phase, primary Si, Cu-rich phases such as CuAl_2_ or Cu-Al-Si-Mg, and Al-Si eutectic.

The element Be is added both to alter the morphology of insoluble Al-Mg-Fe-Si phase to nodular form and to modify the chemistry of insoluble phase to exclude magnesium, thereby ensuring its availability for precipitation hardening. Since it is a heat-treatable alloy, the strength, fatigue, and corrosion resistance are enhanced significantly after heat treatment using T6 temper. Further, the melt is treated with grain refiners like Al-3Ti-B, Al-5Ti-B, or Al-Ti-C master alloys [32]. Melt modification along with heat treatment changes the morphology of silicon from a coarse acicular to fibrous one, due to which its properties are enhanced.

The strength and wear resistance of A357 alloy are increased with the addition of ceramic particulates. To this end, efforts have been made to reinforce A357 with graphite fibers, TiB_2_, and Al_2_O_3_ particles to modify the modulus, as well as electrical and thermal properties [33,34].

### 2.3. Dual-Particle-Size (DPS) Composites and Their Advantages

It is well known that the properties, such as wear resistance, of MMCs largely depend on interfacial bonding, matrix properties, type of reinforcement, particle weight, volume fraction, and particle size. Few researchers have reported that the MMCs containing smaller-size particles have shown higher wear resistance when compared to the MMCs with large-size particles. Taking a cue from this, several research investigations have been carried out to study the effect of dual particle size (DPS) on the mechanical and tribological behavior of MMCs. The size difference between these particles will be quite large, that is, for example, the large particle size will be several hundred micrometers, while the smaller particle size will be a few micrometers, or in some case it will be of submicron size. The most explored particulates for DPS composites are SiC (2/163 µm), TiC (2 µm/100 nm), B_4_C (3 µm/50 nm), Al_3_BC (5/300 nm), Al_2_O_3_ (100/150 µm), and ZrSiO_4_ (20/125 µm). The idea of using two different sizes of particles in composites is to get the benefit from their size difference. The addition of differently sized particles has a significant effect on grain size and its orientation. In addition, the intensities of texture components change. For instance, during wear testing, the larger particles not only carry the greater amount of load applied on the composites, but also protect the smaller particles from being removed. On the other hand, small-size particles not only help in reducing the plastic flow by minimizing the plastic deformation, but also aid in preventing the adhesive transfer of material during wear test. These dual-size particles have shown that they can bring coordinated improvements in mechanical properties such as strength, ductility, and hardness. Furthermore, an important property, such as creep resistance, of composites will also improve by 3 to 6 times for dual-particle-reinforced composites when compared to single-reinforcement composites and alloys. Most of the dual-size particles were used to reinforce pure aluminum, Al-3Cu, Al2124, Al6061, Al6063, LM13 piston alloy, magnesium, and copper.

### 2.4. Applications of Al-Si Alloys

There has been steady growth in utilizing aluminum alloy castings for both aircraft and automotive applications after World War II. During this time, the United States of America made P-51 aircrafts that had the fuselage entirely made up of aluminum for weight reduction. Furthermore, a decrease in the weight of the P-51 engine was achieved by employing Duralumin alloy to make the cylinder block, pistons, and crankcase. In the present day, Al-Si alloys are employed in making complex substructures using the investment-casting process for the aerospace industry. For example, the winglet substructure made of F357 alloy for the Embraer Phenom is made up of a single block. The rear frame avionics bracket and tail rotor gearbox are made up of D357 alloy, using precision sand casting technique. Furthermore, Al-Si alloys are used for making powertrain components in the automotive industry by replacing gray cast iron. In making a V8 engine block, if Al-Si alloy is used, then the overall weight of block is 32 kg, whereas if gray cast iron is used, then it weighs 68 kg. Therefore, there is a considerable amount of weight reduction by opting for Al-Si alloys in place of gray cast iron. In addition, lot of efforts have been dedicated by the North American automotive industry to replace iron engine blocks and cylinder heads with cast Al-Si alloys. For example, Chrysler and Ford use Al-Si alloy for making cylinder heads (C351) using a semi-permanent casting technique. On the other hand, General Motors uses aluminum alloys to produce wheel rims by permanent casting. Chevrolet Corvette uses Al alloys for making engine blocks by sand casting process. Many minivans, cross-over vehicles, cars, and trucks employ aluminum alloys for making door handles or roof brackets [35,36].

### 2.5. Potential Applications of Al-Based MMCs

**Figure 2:**
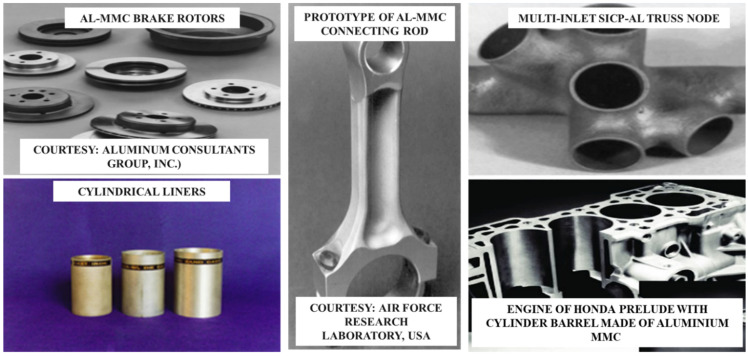
Applications of Al-based MMCs in automotive industry [38].

Table 4 lists the applications of Al-based MMCs used in different industries (automotive, aerospace, sports, and construction). Figure 2 shows the applications of Al-based MMCs used in automotive industry.

**Table 4:** Applications of Al-based MMCs [37].

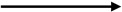	Property? Weight Reduction	Wear Resistance	Stiffness	Thermal Conductivity	Tailorable CTE	Corrosion Resistance	Corrosion to Radiation	High Strength
Benefits								
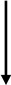								
Potential and Existing Applications								
**Bearings**	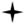	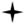				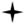		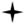
**Brake rotors**	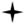	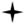						
**Engine cylinder liners**	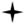	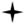						
**Pistons**	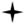	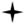						
**Worm gears**	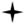	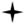						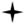
**Aircraft skins**	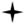							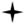
**Bicycle frames**	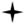		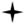					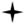
**Electronics packaging**	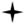			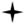	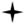		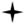	
**Ground vehicles**	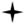							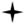
**Medical implant**	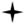					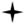		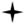
**Sea vehicles**	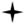		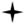			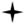		
**Space structures**	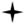		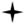	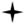			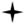	
**Transmission components**	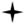	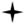				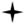		
**Turbine engine components**	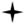	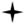						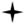

## 3. Fabrication Techniques and Factors of Al-Based MMCs

### 3.1. Methods

Metal-matrix composites offer promising mechanical and physical properties for various applications, yet their applicability has been limited. Processing MMCs using sophisticated fabrication techniques results in a high cost of the end product and is therefore one of the greatest barriers to expanding the applications of MMCs. By developing good and low-cost fabrication techniques, one can improve the commercial applicability of MMCs. Generally, the processing of MMCs is divided into three main classes: (i) liquid-state, (ii) solid-state, and (iii) gaseous-state processing. A brief discussion on all three processing routes and their advantages is documented below with some examples [3,39,40,41].

#### 3.1.1. Liquid-State Fabrication 

**Figure 3:**
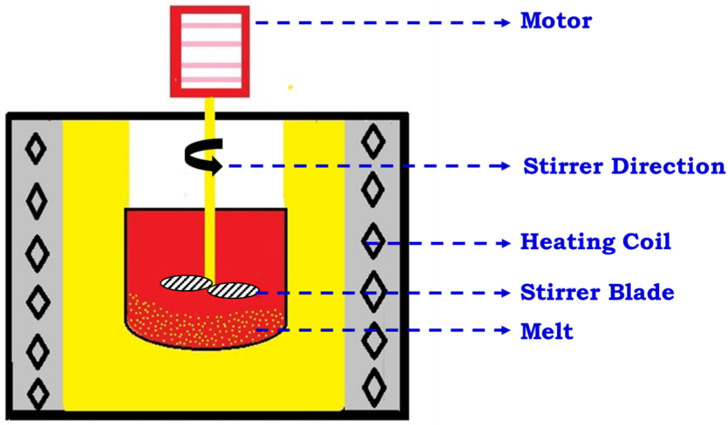
Schematic diagram of stir-casting technique.

In this type of fabrication technique, the reinforcement is added when the metal matrix is in a liquid state. This technique is capable of producing large-scale products at a faster rate and has been proven to be more beneficial for low-melting-temperature metals such as aluminum and magnesium. This technique can be further divided into four important categories, namely stir-casting/dispersion, squeeze-casting/pressure infiltration, in situ, and spray processes. Each of these fabrication techniques have their specific applications. No single technique can be considered to be universally applicable for all alloys, casting sizes, and final components. The present work involves casting of MMCs using the stir-casting technique, which is one of the most widely used techniques for fabricating MMCs. Companies such as Duralcan use this technique to produce aluminum composites reinforced with particulates. Hence, only the stir-casting technique along with different liquid-state fabrication techniques are discussed herein. In the case of stir casting, the casting of MMCs is done in semisolid conditions, which means that the temperature is between liquidus and solidus. A simple schematic diagram depicting the stir-casting method is shown in Figure 3, where a mechanical stirrer is provided in the middle of the furnace for creating agitation in a semisolid slurry. Here, the molten metal is agitated vigorously and allowed to cool down to semisolid state. The agitation is done by using a mechanical stirrer whose material is entirely different from those that are being cast. The advantage of continuous agitation is that it prevents a rise in the viscosity of the slurry and the breaking of solidifying dendrites into spheroidal particles [39,40,41]. During agitation, the reinforcement, such as particulates and short fibers or whiskers, is added into the semisolid metal.

The reinforcement, which is in an agitated condition, does not agglomerate or flow on top of the semisolid slurry; instead, it is trapped by the solid in this slurry with uniform dispersion. Here, the stirring or agitation of the semisolid slurry is very important as it breaks the agglomerates of particulates. Regardless of the lack of wettability of ceramic particulates with metal matrix, this technique helps in entrapping them in the semisolid slurry so that they are uniformly dispersed. The continuous agitation helps in bonding between both the constituents of the MMCs by bringing them in direct contact. The direct and intimate contact helps in bonding and improving wettability, which improves further with an increase in mixing time. Since the agitation causes a decrease in viscosity, the advantage is that this composite mixture with low viscosity can be directly cast into the required shape. The other advantage of this technique is the absence of shrinkage cavities in the final product, which is mainly because of processing at semisolid conditions where the slurry is nearly in solid state [42].

#### 3.1.2. Solid-State Fabrication

This is another important fabrication technique, in which a high-volume fraction of reinforcement can be incorporated into the metal matrix. In solid-state fabrication, powder metallurgy is the most widely used processing method for the fabrication of metal matrix composites. The reinforcements are mixed with the metal powders using different mixing techniques and consolidated either at room or elevated temperatures. If the powders are consolidated at room temperature, then an additional processing method known as sintering is adopted. In addition to this, secondary processing techniques such as extrusion, rolling, equal-channel angular extrusion, forging, or multi-axial forging techniques are also used. However, such techniques are very expensive and mass production is not possible, thereby restricting them to specific applications. Further, the tooling costs required for the powder metallurgical processing are very high, which also limits its applications [40,41].

#### 3.1.3. Gaseous-State Fabrication

This fabrication technique includes deposition of vapor on the substrate material, with control over the composition of the composite. Two different types of fabrication techniques are the chemical vapor deposition (CVD) and physical vapor deposition (PVD) processes. Here, the control of various factors such as gas mixing, oxygen contamination, and vapor transport are very important for good coating of the composite. However, in the case of gaseous-state fabrication techniques, the availability of metal and the compound target is quite difficult, and deposition rates are slow. In the case of thermal spray techniques, parts with complex shapes are difficult to coat, and most importantly, the starting materials are quite expensive [40,41]

Out of all the processing techniques, the liquid-state fabrication technique is more popular due to its inherent advantages over the solid- and gaseous-state fabrication techniques. Firstly, liquid metal is less expensive when compared to powders, and secondly, the handling of liquid metal is easier and more flexible when compared to that of powders. In addition, a wide variety of shapes can be produced using the liquid-state processing technique, which is not the case with the solid- and gaseous-state fabrication techniques. However, there are some concerns, such as poor control of fabrication parameters and formation of undesirable interfacial products at the interface between matrix and reinforcement. The chemical reactions taking place at the interface can lead to the formation of carbides and oxides, which are highly brittle in nature. Lastly, one needs to consider the economic viability of the liquid-state fabrication technique with respect to its potential for near-net casting [40,41].

### 3.2. Factors Influencing the Mechanical Performance of Composite Materials

The mechanical performance of composite materials depends on (i) interfacial bonding, (ii) orientation, (iii) material, and (iv) wettability. A brief discussion on all these factors is provided below.

#### 3.2.1. Interfacial Bonding

There are different types of bonding between reinforcement and metal matrices, namely chemical, mechanical, and reaction. Generally, a metallic coating such as nickel or copper on the surface of fibers or particulates is applied by electroless technique. The thin layer formed on the surface of the reinforcement tends to form a good adhesion with the metal matrix, thereby increasing the bond strength. On the other hand, reaction bonding occurs when the atoms of the reinforcement diffuse into those of the matrix and vice versa. Mechanical bonding between the reinforcement and matrix is created by etching the surface of the reinforcement in such a way that the increased surface roughness causes interlocking. However, high surface roughness is fraught with the danger of formation of interfacial voids owing to incomplete penetration. When a load is applied parallel to the interface, the mechanical bonding plays an important role, wherein the load is efficiently transferred from the matrix to the reinforcements. At the same time, one should also understand that the mechanical bond alone is insufficient for good interfacial bonding between the reinforcement and matrix. Overall, chemical bond is known to be the strongest bond, and mechanical bond is known to be the weakest [43,44].

#### 3.2.2. Orientation

The orientation of reinforcements plays an important role since the properties along the direction of reinforcements (especially for fibers) are significantly improved. Similarly, in the materials where the fibers are oriented in different directions, the properties such as strength will be high in the direction of fiber orientation. However, in the case of particulates, the orientation does not play a significant role since the properties are the same in all directions, which is contrary to the fiber-reinforced composites. The strength of unidirectional composites is unmatched when compared to that of woven fabric and particulate composites [45].

#### 3.2.3. Material

The strength of MMCs mainly depends on the material of reinforcement. Though the material plays an important role, the application determines the type of reinforcement material. In certain industries such as aerospace, where cost is not the constraining factor, high-strength and high-elastic-modulus fibers based on carbon are used. For applications where both cost and properties are important factors, glass and aramid fibers are utilized. However, for automotive applications, economical materials such as ceramic particulates are utilized. Diamond and nanomaterials such as graphene, which possess higher thermal properties, are used for heat management applications [45].

#### 3.2.4. Wettability

This is one of the important criteria when metal matrix composites are produced by casting with pressure infiltration techniques. The degree of wettability is usually determined by edge angle adjustment of the drop of molten metal on the base of a solid. One should study the surface and interface strains for composite systems to understand wettability. Based on the interface energy and edge angle adjustment, the angle limit for a wettable system is <π/2, while for a non-wettable system, the angle limit is >π/2. One should understand that as the angle decreases, the wettability of a molten metal with its solid base increases. The wetting of molten metal/drop depends on kinetics, which means that it depends on temperature and time. Furthermore, if any alloying element is added to the metal, then the wettability is affected because the surface tension of the melt droplet changes. For example, in infiltration, if the wettability is good, then one can observe a capillary effect of melt in-between the fibers. However, if the wettability is not good, then such an effect is not seen. In the case of the particulate-reinforced MMCs, poor wetting of particles can result in segregation of particles [46].

## 4. Heat Treatment of Al Alloys

The purpose of heat treatment of Al alloys is to achieve the best possible mechanical properties for the desired industrial applications. A sequence of microstructural modifications enhances the mechanical properties based on thermal treatment factors, namely temperature and time [30,47,48].

### 4.1. Temper Designations Used in Heat Treatment

**Figure 4:**
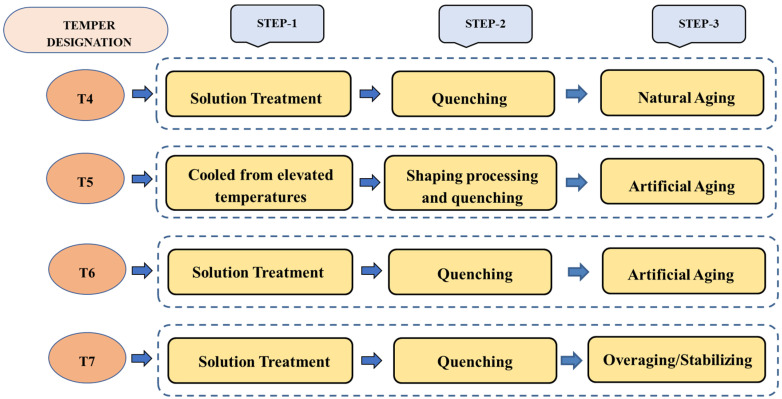
Schematic representation of commonly used temper designations in heat treatment.

The temper designations used for as-cast alloys are F, O, and T, which stand for as-fabricated, annealed, and thermal treatment. Here, the temper designation T is further subdivided into T4, T5, T6, and T7 (commonly used designations), which indicates artificial and natural aging conditions. A schematic representation of the aforementioned temper designations is shown in Figure 4.

### 4.2. Precipitation-Hardening Heat Treatment

In addition to heat treatments such as stress annealing for removing residual stresses, the Al-Si-Mg alloy castings are subjected to different types of heat treatment processes, one of which is solution heat treatment, which is carried out with and without quenching. Heat treatment, which is generally adopted for the enhancement of mechanical properties, starts with homogenization and precipitation hardening. The precipitation-hardening process involves solutionizing followed by quenching and artificial aging [49,50].

(i) *Solutionizing:* Solutionizing aims to obtain a homogeneous solid solution of the material. Here, the alloy is heated to a pre-set temperature to dissolve all the soluble alloying elements and is retained at that temperature for a sufficient time [49,50].
(ii) *Quenching:* The aim of quenching is to keep the alloying elements trapped in the solution and to achieve maximum super-saturation of the alloying elements [49,50].
(iii) *Artificial aging:* Artificial aging aims to reduce the time for precipitating the dissolved impurity or solute phase [49,50].

**Figure 5:**
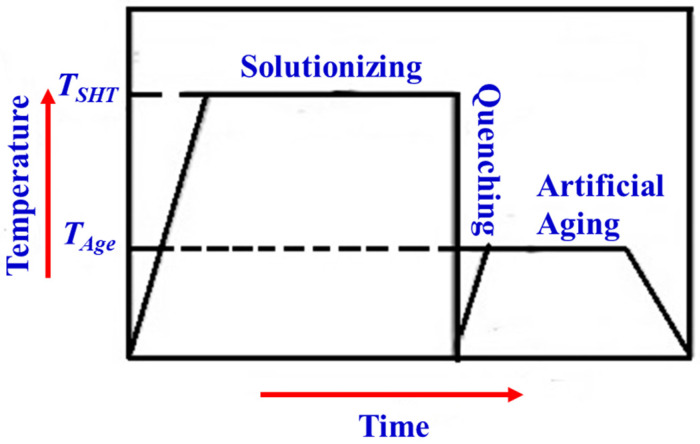
Schematic diagram of precipitation hardening (temperature vs. time diagram).

A schematic diagram of precipitation hardening (temperature versus time diagram) is shown in Figure 5.

For Al-Si-Mg alloys, T6 temper conditions are preferred, which are documented by ASTM standards such as B917 and B91. According to these standards and as documented by several researchers, the solution treatment suggested varies from 450 °C to 560 °C for 4–12 h, and aging temperature ranges from 150 °C to 250 °C for 6–12 h [51,52]. Different combinations of eutectic phases such as Mg_2_Si, Al_8_Fe_2_Si, and eutectic Si are formed with solid solutions.

**Figure 6:**
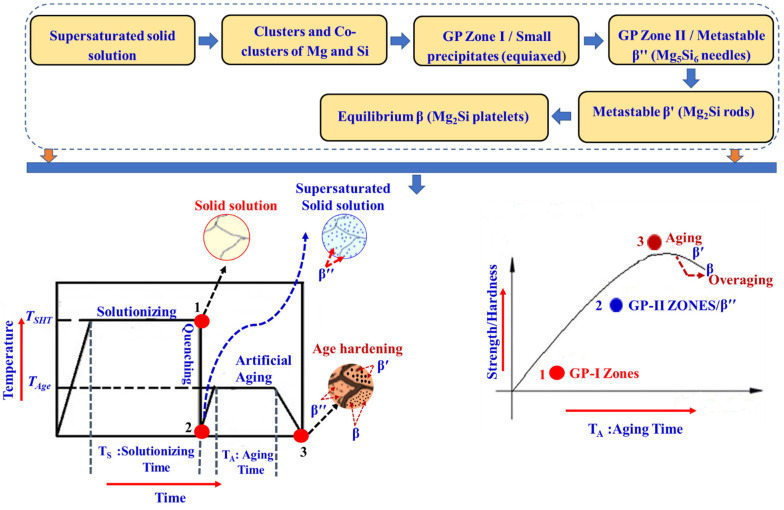
Precipitation sequence of Al-Si-Mg alloy.

Further addition of reinforcements, such as ceramic and carbon particulates, changes the aging sequence and decreases the time required for aging. The aging sequence after the heat treatment process in Al-Si-Mg alloy is shown in Figure 6 [49,50,53,54].

### 4.3. Effect of Heat Treatment on Mechanical Behaviour and Strengthening Mechanisms

Addition of reinforcement to the metal matrix can bring a number of changes in the microstructure of MMCs. Mechanical properties such as strength, modulus, fracture toughness, and creep resistance are increased significantly when ceramic or organic reinforcements are added to the metal matrix. For example, when reinforcement is added to metal matrix, the difference in coefficient of thermal expansion (CTE) can introduce dislocations, especially near the interface region. Apart from dislocations, other defects such as vacancies are found to appear near the reinforcement/matrix region [49,50]. Such defects are capable of affecting the chemical reactions as well as aging kinetics. Aging kinetics of a precipitation-hardenable alloy such as A357 is highly influenced due to the addition of reinforcements, but the precipitation sequence remains the same. The aging kinetics are accelerated in the presence of reinforcement by virtue of the enhancement in dislocation density. These dislocations act as heterogeneous nucleation sites for precipitates during natural ageing treatment [53,54]. In addition, the hardness and strength enhancement greatly depends on the type of precipitates formed and their respective size. However, it is necessary to have controlled reactions at the interface to obtain strong bonding between the matrix and reinforcement. A thick interaction region will have detrimental effect on the properties of composites. A strong and continuous interfacial bond will ensure transfer of applied load from the matrix to the hard and strong reinforcements. Here, the interfacial bonding does depend significantly on the wettability of the reinforcement by the metal matrix. In the case of MMCs produced by casting technique, the normal dendritic structure is entirely different when compared to unreinforced metals. The morphology of the dendritic structure is completely controlled by fiber or particle dispersion, due to which grain refinement is achieved. On the other hand, the secondary phases prefer to appear at the interface region since most of the chemical and mechanical interactions occur here. Overall, such high densities of defects will alter the various properties of MMCs, which can be tailored based on reinforcement size, morphology, and weight fraction [20,55,56].

## 5. Mechanical and Tribological Properties of Al-Based MMCs

This section presents a critical review of the published literature relevant to the present work. Firstly, the prior work on the mechanical properties of Al-based MMCs is discussed. Next, a review of the existing work on their tribological properties is presented. Finally, a subsection on the dual-particle-size reinforced composites is included.

### 5.1. Mechanical Properties of Al-Based MMCs

The mechanical behavior of materials plays a significant role in the development of new materials. There are different techniques to improve the mechanical properties, which include the addition of reinforcements, heat treatment, and secondary processing techniques. This section summarizes the research work that has been carried out by several researchers on single-particle-size (SPS) reinforced Al-based MMCs, and also the influence of heat treatment on their mechanical properties.

First, we look at the effect of SiC-reinforced A357 composite fabricated by stir casting and hot isostatic processing (HIP) techniques. The SiC particulate chosen had an average particle size of 30 µm, and about 15 vol.% SiC was reinforced to A357 matrix. This work reported the yield (163 MPa) and nominal strength (367 MPa) of A357 alloy in as-cast condition. In contrast, the same strength values determined for the A357/SiC composite were much lower. Furthermore, the values of these two properties of the A357/SiC composite obtained from the HIP process were also lower, at 96 and 214 MPa, compared with the base alloy A357 (121 and 274 MPa, respectively). The reduction in properties of the reinforced composites were attributed to the formation of brittle phases and high levels of porosity [57]. Churyumov et al. [58] studied the mechanical properties of Al-Si-Mg composite reinforced with SiC particles fabricated by stirring and pressure crystallization techniques. Different percentage (5, 10, and 15%) of SiC particles of 40 µm size were added to the Al-Si-Mg matrix. The yield strength of Al-Si-Mg alloy and composites with different SiC content (5, 10, and 15%) were 295, 310, 325, and 330 MPa, respectively. The improvement in strength was attributed to the pressure crystallization process as it enables uniform dispersion of SiC particles when compared to that of cast composites. In addition, the pressure crystallization process helped achieve better bonding between SiC particulate and Al-Si-Mg matrix.

Electromagnetic frequency’s effect on the mechanical behavior of A357 composite reinforced with SiC nanoparticles was investigated by Mohammadi Badizi et al. [59]. The nanocomposites were produced using an electromagnetic stirrer fixed in a resistance furnace, and the entire set up was placed in a vacuum chamber at 750 °C. With an increase in the frequency of electromagnetic stirring, the grain size of both A357 alloy and the nanocomposite was found to decrease. The nanocomposites exhibited higher hardness (60 BHN) when compared to that of A357 alloy (55 BHN). The yield strength (YS) and ultimate tensile strength (UTS) of A357 alloy were 79 and 119 MPa, respectively. A357/SiC nanocomposite showed higher strengths of 120 and 188 MPa, respectively, for 60 Hz electromagnetic stirring. The homogenous dispersion of hard ceramic nanoparticles was attributed to be primarily responsible for an increment in the hardness and strength of nanocomposites. Ajay Kumar et al. [60] also reported that both YS and UTS will increase due to the incorporation of graphene nanoplatelets (GNPs) in the friction stir processing (FSP) of squeeze-cast A356 alloy. Kandemir [61] employed the ultrasonic cavitation method for processing A357/SiC composites and evaluated their microstructure and mechanical properties. The A357/SiC composite exhibited higher hardness (73 HV) and tensile strength (198 MPa) when compared to that of A357 alloy, which exhibited a hardness of 60 HV and tensile strength of 138 MPa. The author attributed this increase in strength and hardness to a reduction in the grain size of the composites, thereby assisting the Orowan mechanism. On the other hand, A357/SiC composite had little effect on the load-bearing mechanism. Tekmen and Cocen [62] studied the effect of heat treatment on Al-Si-Mg/SiC composite developed by compo-casting technique followed by extrusion. All unreinforced and reinforced materials were solutionized at 530 °C for about 2 h followed by artificial aging at 175 °C for about 48 h. For Al-Si-Mg alloy and 20 wt.% SiC-reinforced composite, the hardness obtained after peak aging time varied with the cold working plastic strain (4, 10, 25, and 50%). For strain rates of 4 and 10%, the peak aging time was 8 and 7 h, and the hardness of the alloy and composite was 106 and 120 HV, respectively. The increase in hardness was due to grain refinement and transformation of coherent and semi-coherent precipitates. Li et al. [63] studied the effect of heat treatment on an Al-Si-Cu-Mg/SiC composite developed by electromagnetic stirring technique. The composite was solutionized at 520 °C for about 6 h and water-quenched at room temperature, followed by artificial aging at 175 °C for about 6 h. The microhardness and UTS of the composite before heat treatment were 87 HV and 239 MPa, respectively, while after T6 heat treatment, the hardness and strength increased to 102 HV and 274 MPa. Al_2_Cu-based needle-like precipitates of 100 nm length, formed after heat treatment, were mainly responsible for the improvement in the mechanical properties.

During the investigation on the mechanical properties of the Al-Si-Mg-Ti alloy containing TiC nanoparticles and fabricated by the selective laser melting (SLM) method, it was noticed that this alloy showed an increment in its mechanical properties such as UTS (nearly 562 MPa) and elongation (about 8.8 % until fracture) due to the grain refinement effect, as well as the Orowan/load-bearing mechanisms of strengthening the alloy. Gao et al. [64] also employed the SLM technique along with the ultrasonic vibration method to fabricate TiN/AlSi10Mg nanocomposites with the TiN nanoparticle content varying from 0 to 6 wt.%. The authors indicated that the specimens manufactured from the additive manufacturing route showed high ductility (nearly 7.5%), high tensile strength (nearly 492 MPa), and microhardness of about 157 HV. The mechanisms that caused the increment in the strength of the nanocomposites were the Orowan/load-bearing, CTE, and Hall–Petch strengthening mechanisms. Furthermore, the reduction in porosity, grain size refinement, and dislocation-slip resistance (produced by the dispersed TiN nanoparticles) yielded higher ductility in the composites [65]. Cocen et al. [66] reported the age-hardenability of Al-Si-Mg/SiC composite fabricated by compo-casting technique. Composite samples were solutionized at 530 °C, quenched in water, and aged at two different temperatures of 150 and 175 °C. The composite when aged at 150 °C showed a peak aging time of 180 min and hardness of 33 HB, and when the same composite was aged at 175 °C, the peak aging time was 100 min and the hardness was 16 HB. The decrease in hardness after 150 °C aging temperature was attributed to low hardening efficiency and depletion of Mg content to form Mg_2_Si precipitates. The work showed that the critical aging temperature was highly dependent on both the microstructure of the matrix and the amount of Mg_2_Si precipitates. Bloyce and Summers [67] employed the squeeze-casting technique to process A357/SiC Al-based MMCs and carried out heat treatment at two solutionizing temperatures (525 °C and 540 °C) and two aging temperatures (170 °C and 160 °C).

From the studies on static and dynamic mechanical properties, the authors concluded that solutionizing at 540 °C and aging at 160 °C resulted in higher mechanical properties for both the A357 alloy and the A357/SiC composites.

In a study of the precipitation kinetics of in situ Al-Si-Mg composite reinforced with TiB_2_ particles, a composite with 5 wt.% percentage of TiB_2_ was produced using the salt reaction technique. This composite was solutionized at 540 °C for about 8 h followed by hot water quenching. Artificial aging was conducted at 170 °C for a duration of about 7 h, followed by mechanical testing. Upon heat treatment, the hardness of the alloy and the composite increased from 62 to 95 HB and from 72 to 105 HB, respectively [68]. Similarly, the tensile strength of the alloy and the composite increased from 98 to 155 MPa and from 114 to 212 MPa after T6 heat treatment, respectively. The strengthening of the aged composites was attributed to the modification of eutectic Si phase and better dispersion of TiB_2_ particles. Furthermore, related to the precipitation-strengthened composites obtained from the cast eutectic alloy in the Al-Si-Mg system, the nanosized SiC powder was the strengthening phase [69]. Here, the yield stress (320 MPa) and tensile strength (350 MPa) were noticed in the composite ribbons with high strength. In addition, the high-temperature strength can be maintained up to a temperature of 300 °C. Wang et al. [70] studied the effect of T6 heat treatment and addition of Sr on the mechanical properties of A356/TiB_2_ composites. The composites were heat-treated, wherein solutionizing was carried out at 540 °C for about 12 h, followed by cold water quenching. The final step was artificial aging, which was conducted at 155 °C for about 10 h. The YS and UTS of A356 alloy before heat treatment were 97.5 and 151.5 MPa, respectively, while those of the composite were 109 and 164.5 MPa, respectively. After heat treatment, the YS and UTS of A356 alloy increased to 208.1 and 263.1 MPa, respectively, while those of the composite increased to 255.5 and 312.5 MPa, respectively. The formation of fine-size Mg_2_Si precipitates in the Al grains caused an increase in strength following heat treatment. Kumar et al. [71] investigated the age-hardening effects of Al-Si-Mg hybrid composites reinforced with ZrSiO_4_ and Al_2_O_3_ particulates. The composite fabricated using the casting technique was solutionized at 540 °C for about 3 h, followed by cold water quenching. Then, aging was done at 170 °C for different time durations ranging from 0 to 480 min. Compared to zircon-reinforced composites, the one reinforced with alumina showed a high hardness of 118 HV for an aging duration of 360 min. The hardness increased with an increase in aging time for up to 360 min, following which it started to decrease. Formation of Mg_2_Si precipitates in the matrix material led to an increase in hardness.

In the case of the age-hardening behavior of A356/TiB_2_ composites fabricated by thixoforming, solution treatment was conducted at 540 °C for a time duration of 8 h, and quenching was perfomed in cold water. Soon after quenching, the samples were aged at 160 °C for a time duration varying from 0 to 24 h. The samples were air-cooled to room temperature after artificial aging and subjected to microstructure analysis. A356 alloy showed a coarse dendritic structure, while after the addition of TiB_2_ and heat treatment, the structure was transformed into a fine equi-axed structure. This study reported that the time taken for peak aging in the case of A356 alloy was 12 h, while in the case of composites, it was 8 h. The peak aged samples were subjected to transmission electron microscopy studies, which revealed the formation of GP II zones and Mg_2_Si precipitates. The sizes of these precipitates were in the range of 10 to 20 nm, with most of them located at the grain boundaries [72]. Samuel et al. [73] studied the effects of heat treatment on SiC-reinforced Al-Si-Mg composites. Solution treatment was carried out at 538 °C for about 8 h, while the quenching was performed in warm water at 60 °C. The artificial aging was at 155 °C for 5 h, followed by air-cooling to room temperature. The microstructure showed the formation of Al_4_C_3_, Al_2_O_3_, and MgAl_2_O_4_ spinels at the SiC and Al interface. Formation of Mg_2_Si precipitates were also seen in the microstructure after T6 heat treatment. Abdulwahab et al. [74], in their work, studied the effect of heat treatment on an Al-Si-Mg/melon ash composite developed by stir-casting technique. Here, the heat treatment was carried out with a solution treatment conducted at 540 °C for about 1 h, followed by warm water quenching at 65 °C. Aging was conducted at 180 °C for about 2 h, and it was later air-cooled to room temperature. The heat treatment process resulted in the formation of Mg_2_Si precipitates along with an Al_2_Mg_3_ intermetallic compound. On the other hand, plates corresponding to Al-Si-Mg were also seen in the aluminum matrix. Menargues et al. [75] reported the heat treatment of semisolid-processed Al-Si alloy. In this study, the alloy was subjected to T6 heat treatment, with solution treatment less than 30 min, as mentioned in the standard procedures. Solution treatment of the alloy was carried out at three different temperatures, 520, 530, and 540 °C, with varying times ranging from 5 min to 5 h. After solution treatment, the alloy was quenched in liquid media, such as water that was kept at room temperature. Then, artificial aging was conducted at 160, 170, and 180 °C with the time duration ranging between 1 to 5 h. Here, aging was necessary to decompose the supersaturated solid solution, and then, in turn, to form fine precipitates. Microstructural analysis showed the formation of π-AlFeSiMg, needle-shaped β-AlFeSi (intermediate phase), and dark-colored Mg_2_Si phases.

In a study on the effect of heat treatment on the impact toughness of aluminum alloy A356, the alloy samples were solution-treated for 540 °C for about 6 h, followed by quenching in water. The artificial aging process was conducted at 155 °C for a time duration of 4 h, and it was then air-cooled. The time gap between the solution treatment and artificial aging was less than 30 s, and this also involved quenching in water. The morphology of eutectic silicon was changed from plate to rod, and then to spherical shape after heat treatment. In addition, the coarse α-Al dendrites were transformed to fine equi-axed grains with fine fibrous silicon particles following heat treatment [76]. Ebenezer et al. [77] investigated the mechanical properties, such as UTS, yield strength, and hardness, of Al_7_SiMg/rice husk ash (RHA) MMCs, where the composites were fabricated by using the stir-casting method. Electrodeposition of nickel was carried out for the base alloy as well as the MMCs through the electrolytic watts bath with conventional stirring. Higher values of the aforementioned mechanical properties were noticed with an increase in the RHA content [77]. In an identical manner, Senan et al. [78] also observed that the mechanical properties such as ductility, hardness, yield, and tensile strength of the A356/hybrid MMCs will be improved due to the rise in the reinforcements (that is, Al_2_O_3_ and B_4_C particles) in terms of weight percentage. In a more recent work by Sakthi Sadhasivam et al. [79] in 2022, the stir-casting technique was employed to manufacture an Al-Si-Mg alloy with ZnO microparticles as the reinforcement. The authors indicated that the mechanical properties and the various stress/stress ratio parameters will be enhanced due to an increment in the ZnO particles, in terms of weight percentage (that is, 3, 4.5, and 6%), in the resulting composite.

### 5.2. Tribological Properties of Al-Based MMCs

The interaction of two sliding bodies changes when reinforcement is added to one of them. The influence of these interactions can be seen in the coefficient of friction and the wear rate. This is because when two different pure metals or alloys are subjected to a sliding motion, the coefficient of friction and wear rate are very high and can sometimes lead to seizure of the surfaces. On the other hand, significant changes in the microstructure of metals or alloys due to the addition of reinforcement can result in different friction coefficients and wear behavior. Numerous studies have been devoted to reducing the coefficient of friction and wear rate by adding alloying elements, reinforcements, and microstructure modification in the surface region. This sub-section reviews the research carried out on understanding the tribological behavior of single-particle-size (SPS) reinforced Al composites. The review also includes the effect of heat treatment of Al-based MMCs on their tribological properties.

Leonard et al. [80] reported dry sliding wear of SiC-reinforced A357 composite that was developed by casting technique. The 30 vol.% SiC-reinforced A357 composite was subjected to a tri-pin-on-disc testing machine with grey cast iron and 180 HV_10_ as the counterface. At a load of 6 N, the alloy exhibited a high wear rate of 169.6 × 10^−6^ mm^3^/Nm, while the composite exhibited a low wear rate of 3.1 × 10^−6^ mm^3^/Nm. At a load of 74 N, the composite exhibited a high wear rate of 176.8 × 10^−6^ mm^3^/Nm, while the A356 alloy exhibited a lower wear rate of 89 × 10^−6^ mm^3^/Nm. At lower loads, the dominant wear mechanism was oxidation, with some contribution from three-body abrasion. At higher loads, adhesive wear was more predominant, with detachment of large metallic sheets due to subsurface cracking, and thus the composite exhibited a higher wear rate. Zulfia et al. [81] reported the wear testing of Al-Si-Mg/SiC composite fabricated using the stir-casting technique. The test was conducted as per the Ogoshi method, with an applied load of 12.6 kg, sliding speed of 1.97 m/s, and sliding distance of 400 m. The wear rate of the composites was found to decrease with an increase in SiC content from 2 to 15% volume percentage. Al-Si-Mg alloy showed a 0.022 mm^3^/m wear rate, while the composite with 15% SiC showed a 0.004 mm^3^/m wear rate. The microstructure showed the formation of MgO.SiO_2_ and MgAl_2_O_4_ spinels at the SiC and Al interface, which offered wear resistance to the composite. The spinels that formed also ensured minimal SiC particle pull out during the wear test, thereby avoiding severe wear.

The wear behavior of A356-reinforced SiC composites with sliding against grey cast iron was analyzed. The composite with 25% weight percentage of SiC was produced by stir casting and later heat-treated by following T6 temper conditions. At 40 and 60 N loads, the volume loss of cast iron was higher than that of the A356/SiC composite for all cases of sliding velocities. Similarly, at sliding velocities of 2.5 and 3.7 m/s, the volume loss of cast iron was significantly higher than that of the composite for all cases of load [82]. On the other hand, the friction coefficient of A356/SiC composite at 2.5 and 3.73 m/s sliding velocities and under varying load was found to be significantly higher (0.57–0.64) than that of cast iron (0.29–0.39). Based on the high coefficient of friction, the authors recommended the composite for brake rotor applications. Rahimipour et al. [83] reported wear studies on an A356/Al_2_O_3_ composite produced by compo-casting. The cast composite was heat-treated, with solutionizing being carried out at 545 °C for about 4 h, followed by aging at 155 °C for about 6 h. The weight loss of the composite was found to increase with an increase in the applied load from 5 to 30 N. For a load of 5 N, the weight loss was 1.8 mg, while at a load of 30 N, the weight loss was 5.8 mg. Similarly, with an increase in the sliding distance and at a constant load of 10 N, the weight loss was found to increase. The worn surface observed for 10 N load tests was composed of deep continuous grooves and extensive plastic deformation. In the case of a higher specific load, the debris morphology was plate-shaped and had sharp edges, which imply that adhesive wear was predominant. Cam et al. (2016) reported the wear properties of A356 in situ composite reinforced with TiAl_3_ and fabricated using mechanical alloying and powder metallurgy techniques. Here, the wear test was conducted at room temperature and under dry sliding conditions at a load of 30 N and sliding speed of 1 m/s. The weight loss of the composite increased with an increase in sliding distance from 400 to 2000 m. The wear rate of the composite reinforced with 8 wt.% Ti was less than that of the composite reinforced with 4 wt.% Ti. This decrease in coefficient of friction was attributed to the higher amount of intermetallic phases, which play a big role in increase in hardness. For different Ti contents, the coefficient of friction was evaluated, and it was found that the coefficient of friction decreased with an increase in Ti content from 4 to 8 wt.%. The composite with 4 wt.% Ti exhibited the highest weight loss and showed extensive plastic deformation when examined under electron microscope. Sam et al. [84] reported tribo-mechanical studies on an LM25 composite reinforced with TiB_2_, WC, and ZrO_2_ and produced by squeeze-casting. The cast composite was solutionized at 520 °C for about 8 h, followed by aging at 165 °C for 4, 6, and 8 h. The wear tests were conducted as per the RSM model with applied load, sliding speed, and distance ranging from 10–50 N, 1–4 m/s, and 500–2500 m, respectively. Optimal results were obtained by solutionizing at 520 °C for 8 h, followed by quenching and aging at 165 °C for 8 h. The worn surface observed for the 50 N test load had deep grooves and extensive plastic deformation. In another work related to LM25 (Al-Si7-Mg)/WC MMCs [85], a major enhancement in the hardness and tensile strength properties was noticed upon aging the LM25 alloy at 150 °C. In addition, the wear strength of the MMCs with 6 and 9 wt.% WC was improved by up to 30% when compared with the unreinforced specimens.

In a study on the tribo-mechanical behavior of A359 composite reinforced with TiB_2_, TiO_2_, and TiC and produced by modified stir-casting, the cast composite was solutionized at 540 °C for about 10 h, followed by aging at 155 °C for 10 h. A linear reciprocating wear test was carried out with applied load and sliding distance ranging from 15 to 55 N and from 500 to 2500 m, respectively [86]. A359/TiB_2_ composite exhibited the least weight loss when compared to A359/TiO_2_ and A359/ TiC composites. The worn surfaces of T6-treated A359 composites displayed mild plastic deformation, compared to the severe plastic deformation of untreated composites. Padmanaban et al. [87], in 2020, produced a rheo-die-cast (RDC) Al-Si-Mg alloy and RDC Al-Si-Mg/SiC_p_ composites, where the SiC_p_ content varied from 5 to 20 vol.%. There was an enhancement in the hardness and wear resistance in the RDC samples. Furthermore, the wear resistance in the composite with 20 vol.% SiC_p_ was higher by three times compared to the samples without the presence of SiC_p_ reinforcement. The improved hardness of the reinforced samples was also noticed in their work. Vuddagiri and Ravisankar [88] investigated the tribological behavior of an Al-Si-Mg alloy reinforced with molybdenum disulfide (MoS_2_) and alumina (Al_2_O_3_) particles. The resulting composites were manufactured through the stir-casting method. The process parameters considered in their work were the weight percentages of the reinforcements, sliding velocity, and load. The authors found that both the friction coefficient and wear rate were reduced upon increasing the sliding speed, whereas both of these properties were increased due to the higher load. In other words, in comparison with the base alloy, an improved resistance to wear and lower coefficient of friction were shown by these hybrid composites. In an identical work by Balasubramaniam et al. [89] on fly-ash-reinforced aluminum MMCs consisting of different weight percentages of silicon, as the sliding velocity and the applied load increase, the wear rate decreases and increases, respectively. In addition, the tensile strength and hardness of the composites were enhanced with an increase in the Si content [89]. Furthermore, scanning electron microscope (SEM) examination of the composite surfaces revealed that a combination of abrasive and adhesive wear was the principal wear mechanism at a lower load, while at a higher load, adhesive wear was the predominant mechanism of wear [88]. Usman et al. [90] fabricated A356 alloy (Al-Si-Mg)-based composites through the stir-casting approach with locust bean waste ash (LBWA) particles as the reinforcement. Mechanical properties such as tensile strength, hardness, and impact energy were considered in their study. In addition, the wear behavior was examined by performing a wear test under dry sliding conditions on ball-on-disc tribometer equipment. The authors indicated that the presence of 10 wt.% LBWA particles in the composites will provide the highest tensile strength, hardness, and wear resistance. Anilkumar et al. [91] fabricated aluminum metal-matrix composites (AMMC), wherein the Al-14.2Si-0.3Mg alloy was reinforced with coarse TiC particulates of 10 wt.% content. The MMCs were subjected to solutionizing treatment at a temperature of 525 °C for 12 h and were then quenched in water to reach room temperature. The resulting composite was age-hardened at various temperatures and times for improving its wear properties. A (pin-on-disc) tribometer setup was used for studying the wear properties of these composites. Changes in their wear properties were noticed upon increasing the aging time, aging temperature, and applied load. The optimum wear resistance of the composites was noticed under the conditions of 30 N (applied load), 155 °C (aging temperature), and 5 h (aging time). In addition, SEM images showed a reduction in the formation of wear debris and shallow grooves with an increase in the applied load.

### 5.3. Dual-Particle-Size (DPS) Composites

Some recent studies were carried out on reinforcing metal matrices with dual-size (bimodal-sized) particulates. The composites reinforced with dual-size particles have shown better strengthening and wear-resistance properties compared to single-reinforcement composites. Out of the two different sized particles, the larger-size particles contribute to improvement in hardness and wear resistance, while the small-size particles contribute to improvement in strength. This section summarizes the research carried out on dual-particle size (DPS)-reinforced Al-based composites, including their mechanical and tribological properties. Kheirifard et al. [92], in their work, used Ni-P-coated Al_2_O_3_ and SiC particles of two different sizes, namely 170 and 15 µm, respectively, to reinforce the A356 alloy. The Al_2_O_3_ and SiC particles used were of 99.7% and 99.4% purity, with irregular and angular morphology. The Ni-P-coated-particle-reinforced composites were fabricated using the stir-casting technique and hot rolling. This work reported that compared to single reinforcement, the dual-particle-size-reinforced composite exhibited high strength and hardness. Sadeghi et al. (2018) studied the mechanical behavior of spark-plasma-sintered Al/Al_2_O_3_ bimodal composites. The Al_2_O_3_ particles chosen had an average particle size of 20 nm (smaller particle size) and 10 µm (larger particle size), and different weight ratio combinations (2:8, 4:6, 6:4) were used to reinforce the Al. The hardness of the composites increased with an increase in the ratio of nm- to µm-size particles when compared to that of unreinforced Al. Here, the addition of nanoparticles helped in inducing recrystallization, which assists in grain refinement and increasing the hardness of composites. In addition to this, the particles, especially the nano-size ones, help in the formation of new grains by the particle size nucleation mechanism and also inhibit grain growth. Wang et al. [93] used a pressureless infiltration process to prepare dual-particle-size A356-SiC composites with different SiC sizes (14, 28, 50, and 85 µm). The size dispersal ratios were 50:14 and 85:28. They did not observe any particle damage due to the infiltration process. Furthermore, good bonding was confirmed by the absence of pores. The bending strength of composites was found to increase with a decrease in the particle size. The thermal conductivity of the composite decreased because of the interfacial heat barrier and presence of Fe impurities.

Montoya-Dávila et al. [94] reported on the hardness of bimodal SiC-reinforced Al composites developed by pressureless infiltration process. SiC particles of average sizes 10 µm and 68 µm were used to reinforce Al in the ratios of 1:1, 3:1, and 1:3. The bimodal composites showed hardness of 34 HR, while the unimodal composite showed a value of 40 HR. Similarly, the microhardness of unimodal and bimodal composites were 2339 and 3360 kg/mm^2^, respectively. The bimodal composites required higher stresses for plastic deformation, and this was considered the primary reason for their high hardness. This study concluded that work hardening in the case of bimodal composites was quite higher than that of unimodal composites. Dhandapani et al. [95] studied the effect of dual-particle-size reinforcements (carbon nanotubes and B_4_C particles) on the density and microhardness of Al composites. The size of carbon nanotubes and B_4_C particles were 50–80 nm and 150 µm, respectively, with both having purity close to ~99.5%. The composites were fabricated using the powder metallurgy technique, with sintering being carried out in the temperature range of 420–450 °C. The hardness of the single-reinforcement composite (Al/B_4_C) was 70 VHN, while that of the dual-particle-size composites was 78 VHN. However, with an increase in the CNTs content from 1% to 2%, the hardness decreased to 74 VHN. However, it was still higher than that of the single-reinforcement composite (Al/B_4_C). In continuation, Chandrashekar et al. [96] also incorporated dual-sized B_4_C powders, namely, 44 µm and 105 µm sizes, in A356 alloy by using the stir-casting method. It was observed that the higher content of B_4_C powders in the dual-particle-size composites will increase their tensile strength and hardness by up to 35% and 15%, respectively. Khosroshahi et al. [97] studied the effect of dual particle size on the mechanical properties of A356 composites. The dual-size reinforcements, namely Al_2_O_3_ (~170 µm) and SiC (~15 µm), were used to reinforce the A356 alloy using semi-solid stir-casting and rolling techniques. Dual-particle-size composites showed a fine grain size of 49 µm compared to that of the single-reinforcement composite (SiC) and A356 alloy, which exhibited a grain size of 59 µm each. The effect of grain size was seen on the ultimate tensile strength and yield strength of all materials. For example, the yield strengths of A356 alloy and single- and dual-particle-size composites were 148, 191, and 237 MPa, respectively. Similarly, the ultimate tensile strengths of A356 alloy and single- and dual-particle-size composites was 215, 266, and 302 MPa, respectively. It can be seen that the dual-size-particle composites had very good mechanical properties when compared to both the A356 alloy and single reinforcement composites due to grain growth restriction caused by dual-size particles.

Dual-sized particles are not only used to enhance the mechanical properties of many metal matrices, but also to tailor the thermal properties. Arpon et al. [98] studied the thermal response of aluminum composites reinforced with dual-size SiC particles. The composites were produced using the infiltration technique, with the average size of SiC particles being 16 and 170 µm, respectively, and containing 98.5% purity. The coefficient of thermal expansion of the composite decreased linearly with an increase in the particle content. However, no simple relationship between the bimodal particles and hysteresis was obtained for the composites. Chu et al. [99] studied the thermal conductivity of dual-particle-size SiC-reinforced Al-12Si alloy developed by the infiltration technique. Very high SiC particle contents (56–65 vol.%) were used to produce Al/SiC composites by employing powder injection molding followed by infiltration. SiC particles of average sizes 14 µm, 28 µm, 40 µm, and 70 µm, with 99.5% purity, were used to reinforce Al in different unimodal (28, 40, 70) and bimodal (70:28 µm and 40:14 µm) combination ratios, which were subjected to the laser flash method. The dual-particle-size reinforced composite showed better conduction properties when compared to the single-particle reinforced composites. Apart from the mechanical and thermal properties, the tribological properties of dual-particle-size reinforced Al-based MMCs have also been studied. Bindumadhavan et al. [20] studied the effect of dual-size SiC (47 and 120 µm) on the impact energy and wear rate of Al-Si-Mg composites, which were then compared with the single-particle reinforced composites. The impact energy of dual-particle-size SiC composite (12.2 J) was found to be nearly double that of the single-particle reinforced composite (6.7 J). Furthermore, the wear resistance of dual-particle-size composite was nearly 60% higher than that of the single-particle reinforced composite. The authors concluded that dual-sized composites will improve both the wear resistance and the impact energy significantly. Sharma et al. [100] conducted experiments on dual-size Al-Si-sillimanite (1–20 µm and 75–106 µm) composites. The authors showed that a ratio of 3:1 (between the smaller size particles and bigger particles) resulted in the maximum nano-hardness and minimum wear rates. Prabhu [101] processed Fe-based composites where the Fe matrix was reinforced with bimodal-size dissimilar nature (SiC and SiO_2_) particles through powder metallurgy. The author showed that the addition of bimodal-size particles improves the wear resistance of the composites significantly by comparison with single-size-particle reinforced composites. Furthermore, the author concluded that the formation of a mechanically mixed layer was the main reason for the reduced wear rate in Fe matrix composites reinforced with SiC and SiO_2_.

Maleque and Karim [102] reported the wear behavior of dual-particle-size SiC-reinforced Al6061 composites developed through the liquid metallurgy route. The particles of SiC had an average size of 20 and 80 µm, and the combinations of the weight percentage of dual-particle-size SiC were 7:13 and 13:7. The tribology test was conducted using a pin-on-disc tribotester with a counterface made of steel with 60 R_c_. The composite with a higher content of fine SiC particle size showed the lowest cumulative wear (22 mg), while the one with the highest coarse SiC content showed the highest wear loss (24 mg). However, the coefficient of friction for the higher weight fraction of fine-sized SiC particles was higher (0.57) than that of the higher weight fraction of coarse-sized SiC particles (0.56). The composite with the highest content of fine SiC particle size showed a grooving wear mechanism, while the one with the highest coarse SiC content exhibited a ploughing wear mechanism. Arora et al. [103] studied the influence of dual-particle-size rutile on the wear properties of LM13 aluminum alloy composites. The rutile particles with coarse size (in the range of 106–125 µm) and fine size (in the range of 50–75 µm) were reinforced to LM13 alloy using the stir-casting process. Sliding wear tests were conducted using a pin-on-disc testing machine, with EN32 (of 65 HRC) as the counterface disc. Composites with fine-size rutile particles exhibited a lower wear rate (4.7 × 10^−3^ mm^3^/m) when compared to that of the coarse rutile-particle-reinforced composites (8.4 × 10^−3^ mm^3^/m). Fine-size particles help in increasing the hardness and refinement in morphology of the eutectic silicon. The extent of hardness enhancement and silicon refinement by fine-size particles was higher than that of the coarse-size particles. In both cases, oxidative wear was the more predominant mechanism, followed by plastic deformation. Arora et al. [104] also reported the wear behavior of stir-cast-processed LM13 alloy composite reinforced with rutile particles at high temperatures. The LM13 alloy was reinforced with rutile particles of different sizes, namely 50–75 µm and 106–125 µm, as well as different weight percentages, namely 10 and 15%. Dry sliding wear tests were conducted on a pin-on-disc machine using a sliding distance of 3000 m, sliding speed of 1.6 m/s, and two different loads. The wear rate was found to increase slightly with temperature up to 150 °C. However, at higher temperatures above 200 °C, there was an abrupt increase in the wear rate. In addition, at the higher load of 5 kg, the wear rate was found to decrease above temperatures of 200 °C. Formation of a mechanically mixed layer was said to be the main reason for why the wear rate decreased at this temperature. Furthermore, the fine-size-particle-reinforced composites showed minimal wear loss when compared to that of the coarse-particle-size-reinforced composite. In continuation, Singh et al. [105] studied the wear behavior of Al reinforced with SiC and Al_2_O_3_. The wear test was conducted using a pin-on-disc machine at a constant load of 29.41 N, sliding speed of 239 rpm, and sliding diameter of 80 mm. The design of the experiments was conducted using a Taguchi L9 array to study the significant parameters responsible for the wear of the composites. The authors found that particle size played a prominent role in the wear rate of the composites. The other significant parameters were hybrid solute proportion and vacuum pressure. Furthermore, it was noticed that when compared to the triple-particle-reinforced composite, the dual-particle-reinforced composite displayed better wear resistance. Sharma et al. [106], in their work, studied the influence of dual-size garnet on the wear properties of Al-Si alloy fabricated using the stir-casting technique. The particle sizes of garnet used were 50–75 µm and 106–125 µm, respectively, and about 10–15 weight percentage of garnet were reinforced to Al-Si matrix. The study on the effect of reinforcement revealed that the highest content of fine-particle-size reinforced composite showed lower wear rates than that of the composite with a higher weight fraction of coarse particle size. For the two different weight percentages of 10 and 15%, the maximum wear rate of fine-particle-size composites was about 7 and 5%, respectively, which shows better wear resistance compared to that of the coarse-particle-size composites. At higher loads, deep and continuous ploughing groves were found, indicating abrasion of the composites. Kumar et al. [107] reported the effect of dual-size zircon sand on the wear properties of LM13 aluminum alloy composites developed using the stir-casting technique. The average particle size of zircon sand chosen was 20–32 µm (fine) and 106–125 µm (coarse). Furthermore, the different weight percentage combinations of these particles utilized were 3:12 and 12:3. At a load of 5 kg, the wear rate of the composite with higher fine-size particle content showed a lower wear rate of 7.1 × 10^−3^ mm^3^/m, while the composite with higher coarse-size particle content showed a higher wear rate of 9.6 × 10^−3^ mm^3^/m. The wear rate of both dual-size composites was found to be considerably lower than that of the single-particle composite, whose wear rate was 12.7 × 10^−3^ mm^3^/m. Both dual-particle-size composites showed crack propagation and wear loss of material by delamination at a 5 kg load.

In another work, Kumar et al. [108] reported the high-temperature wear behavior of LM13 reinforced with zircon sand. This composite was fabricated using the stir-casting technique. Two different sizes of zircon sand were used, 20–32 µm and 106–125 µm, in the weight percent combinations of 3:12 and 12:3. The composite with a higher weight fraction of fine particle size showed the highest wear rate at 1 kg load and up to a temperature of 475 K, followed by the composites with a higher weight fraction of coarse particle size. However, at a load of 5 kg, the composite with higher weight fraction of coarse particles showed a minimal wear rate when compared to that of the composite with a higher weight fraction of fine particles. Both the composites showed delamination and abrasive wear mechanisms at temperatures below 100 °C, while oxidative wear was the predominant wear mechanism for temperatures above 150 °C. Kumar et al. [109] also reported the effect of dual-reinforcement zircon sand and SiC on the tribological behavior of LM13 aluminum alloy. A dry sliding wear test was conducted at different temperatures ranging from 50 to 300 °C using a pin-on-disc test machine. The counterface used was a hardened disc made up of EN32 steel with hardness of 65 HRC. For the temperature range of 50 to 200 °C, the wear rate of dual- and single-reinforcement composites was found to decrease. This was attributed to the fact that, at these temperatures, the matrix material expands, due to which the particles are held very tightly. With good interface bonding by mechanical interlocking, the wear resistance was minimal at these temperatures. Here, the dual-particle-reinforced composite showed better wear resistance compared to that of the single-particle-reinforced composite, because SiC refines silicon, while zircon acts as the nucleation site for silicon particles. Lakshmikanthan et al. [110] developed the A357 alloy composite, containing dual-sized SiC particles, through the stir-casting method. Various weight fractions, namely 3% fine + 3% coarse, 2% fine + 4% coarse, and 4% fine + 2% coarse, of two differently sized SiC particles were considered in their work. The wear resistance as well as the mechanical properties, such as hardness, tensile and yield strength of the composites, were primarily studied here. The composites exhibited an enhancement in these mechanical properties when compared with the A357 alloy. More specifically, 4% fine + 2% coarse wt. fraction particles showed the maximum tensile strength, whereas 2% fine + 4% coarse wt. fraction particles displayed increased wear resistance and hardness among the dual-sized particle composites and A357 alloy. The chief strengthening mechanisms identified for such high values of strength were the strengthening of dislocations owing to thermal mismatch and the effective load transfer.

In another closely related work, Lakshmikanthan et al. [55] studied the SiC particle size ratio and aging temperature effects on the aforementioned mechanical properties, as well as wear resistance, of the dual-sized SiC-particle-reinforced A357 composites. The composites again consisted of the same three wt.% combinations of fine- and coarse-sized particles as mentioned previously. However, the composites were fabricated through the (melt-stirring based) permanent mold casting method. For the three different DPS composites, with a constant solutionizing temperature of 540 °C, the aging was performed at 160 °C, 180 °C, and 200 °C (that is, T6 treatment) for 6 h duration. The authors noticed the improvement in the wear resistance and the mechanical properties in the case of the DPS (T6-treated) A357 composites when compared to the (heat-treated) A357 alloy. In addition, the existence of more fine-sized particles in the DPS A357 composites leads to better ductility and strength. In a similar way, the coarser particles’ content will increase their wear resistance and hardness. Table 5 gives the various combinations of dual-particle-size reinforcements used in the literature for making MMCs achieve better properties. In many cases, the reinforcement is the same, but the average particle size will be different, while in some different types of reinforcements, different particle sizes were chosen to reinforce the MMCs.

## 6. Additive Manufacturing of Al-Based MMCs

Here, we include a separate section on the additive manufacturing processes of Al-based metal matrix composites (AMMCs). In particular, the promising selective laser melting (SLM) technique is discussed.

In a review by Pelevin et al. [116] on the fabrication of fully dense (net-shape) parts from aluminum metal-matrix composites (AMMCs) containing alumina (Al_2_O_3_), the authors discussed the key features, results, and advantages of these AMMCs by comparing them with those obtained from conventional methods. For instance, the AMMCs manufactured via the SLM method possessed relatively low strength. However, unlike the AMMCs produced by conventional methods, a significant increase in the relative densities (greater than 99%) as well as the tribological properties and hardness of the AMMCs developed from the SLM technique were noticed, thereby suggesting that this method is favorable for AMMCs with Al_2_O_3_ [116]. Conventionally, in 2003, Shorowordi et al. [117] employed the stir-casting method along with hot extrusion to manufacture AMMCs with reinforcement particles such as SiC, B_4_C, and Al_2_O_3_. Analysis of the fracture surface revealed that the AMMC with B_4_C reinforcement showed improved interfacial bonding [117].

Nalivaiko et al. [118] also considered the SLM method for processing Al-Al_2_O_3_ MMC by adding alumina at 10 wt.%. Different parameters such as laser power, scanning speed and hatch spacing were chosen in their study. The authors implemented the layer’s double exposure with a laser beam [118]. Furthermore, Chang et al. [119] conducted additive manufacturing of composite powder systems involving SiC/AlSi_10_Mg, wherein the effect of different starting particle sizes of SiC was investigated for the production of the in situ AMMCs with Al_4_SiC_4_ + SiC hybrid reinforcement. It was noticed that the SiC particles with finer size aided in the reduction of the coefficient of friction and wear rate when compared with the SLM part developed with coarser SiC particle sizes [119]. In another recent work on the SLM additive manufacturing process, the fabrication or 3D printing of arbitrary structures of aluminum alloys was addressed in detail [120].

Sercombe et al. [121] and Gu et al. [122] conducted a review on the selective laser melting (SLM) of aluminum and AMMCs, to develop intricately formed and efficient metallic parts, comprising metals, alloys, and MMCs. The review also focused on the difficulties associated with the use of aluminum, the rare microstructures obtained by the SLM process, and their influence on the mechanical properties. Additionally, the heat treatment’s effects on the structure and properties are discussed in their work. Lastly, the problems and advantages of manufacturing AMMCs by use of the AM process are also discussed here [121,122]. In continuation, Jue et al. [123] examined the outcome of SLM process parameters on the microhardness, microstructure, and wear of aluminum-based composites containing Al_2_O_3_ particles as the reinforcement. The resulting composite was developed by the SLM technique [123]. However, Wang et al. [124], in 2018, fabricated a TiB2/Al-Cu-Mg-Si composite (heat-treatable type) through the SLM method. The authors observed that Orowan strengthening and grain refinement led to increased strength in the case of a TiB_2_/Al-Cu-Mg-Si composite that was subjected to heat treatment [124]. Prashanth et al. [125] examined the processing of Al–12Si matrix and Al–12Si–TNM composites by SLM process, and detailed structural, microstructural, and wear studies were carried out. From the studies carried out on Al–12Si matrix and the Al–12Si–TNM composites, it was inferred that the composites displayed superior structural properties and the lowest wear rate and that the wear rate rises with an increase in loads.

## 7. Conclusions

A comprehensive literature survey was conducted on the development and processing of Al-based metal matrix composites (MMCs), including their mechanical and tribological properties. In addition, additive manufacturing processes of Al-based metal matrix composites (AMMCs), in particular the promising selective laser melting (SLM) technique, are discussed here.

From this review, it can be seen that the emphasis has been to replace cast iron, which is a traditional material used for aerospace and automobile applications, with MMCs. Furthermore, it is reasonable to draw conclusions that the dual-particle-reinforced composites exhibit better properties than the single-particle-reinforced composites. However, a key question arises with respect to the optimal ratio between the two sizes of particulates that should be used to obtain the optimal combination of mechanical and wear properties. Furthermore, there are other notable findings that emerge from this work. These include issues such as the effect of the addition of dual-particle-size SiC on the mechanical and tribological properties of A357 alloy, as well as the effect of heat treatment on the mechanical and tribological properties of dual-particle-sized SiC-reinforced A357 alloy, which have not yet been addressed to date. Hence, this review indicates the vast scope of this interesting field for present and future researchers.

Significant research attention has been paid to fabricating composites with additive manufacturing techniques, which might not induce problems with homogeneous distribution of reinforcements in matrix material. Furthermore, porosity, segregation, and agglomeration problems can be eliminated with additive-manufactured products. Thin sections can be fabricated, which are often difficult with conventional MMCs. Furthermore, authors can automate the production of MMCs with an additive manufacturing process, which is impractical with conventional techniques. Researchers need to optimize the additive manufacturing parameters and make recommendation to industries for ease of fabrication with better qualities, which will require intense research in the near future.

## Figures and Tables

**Table 1:** Classification of reinforcements [10,12].

Reinforcement Type	Aspect Ratio	Examples of Reinforcements
**Continuous fibers**	>1000	Carbon, glass, boron, SiC, kevlar, steel, wood fibers, carbon nanotubes, Al_2_O_3_, Si_3_N_4_, NbTi
**Whiskers/flakes**	>10	Mica, graphite, BN, SiC, Al_2_O_3_, TiB_2_, Al_2_O_3_+SiO_2_
**Particulates**	1–4	SiC, WC, TiC, B_4_C, TiO_2_, Al_2_O_3_, flash

**Table 2:** Properties of particulate reinforcements [10].

Reinforcement	Crystal Structure	Density (g/cm^3^)	Melting Point (°C)	Elastic Modulus (GPa)	Coefficient of Thermal Expansion (10^−6^ K^−1^)
**BN**	Hexagonal	2.25	3000	90	3.8
**B_4_C**	Rhombohedral	2.52	2450	450	5.4
**AlN**	Hexagonal	3.25	2300	350	6.0
**Al_2_O_3_**	Hexagonal	3.90	2050	410	8.3
**SiC**	Hexagonal	3.21	2300	410	4.9
**TiC**	Cubic	4.93	3140	320	7.4

**Table 3:** Designation of cast Al alloys [28].

S/No	Designations	Alloying Elements
**1**	1xx.x	Unalloyed aluminum
**2**	2xx.x	Al alloyed with Cu
**3**	3xx.x	Al alloyed with Si (traces of Cu, Mg)
**4**	4xx.x	Binary Al-Si
**5**	5xx.x	Al alloyed with Mg
**6**	7xx.x	Al alloyed with Zn (traces of Mg, Cr, and Cu)
**7**	8xx.x	Al alloyed with Sn

**Table 5:** Dual-particle-size combinations used in different studies.

Reference	First Particle Type		Second Particle Type	
	Material	Size	Material	Size
Kheirifard et al. [92]	Al_2_O_3_	170 µm	SiC	15 µm
Sadeghi et al. (2018)	α-Al_2_O_3_	20 nm	α -Al_2_O_3_	10 µm
Montoya-Dávila et al. [94]	SiC	10 µm	SiC	68 µm
Dhandapani et al. [95]	CNTs	50–80 nm	B_4_C	150 µm
Khosroshahi et al. [97]	Al_2_O_3_	170 µm	SiC	15 µm
Arpon et al. [98]	SiC	16 µm	SiC	170 µm
Bindumadhavan et al. [20]	SiC	47 µm	SiC	120 µm
Sandeep et al. (2018)	Al_2_SiO_5_	1–20 µm	Al_2_SiO_5_	75–106 µm
Maleque et al. [102]	SiC	20 µm	SiC	80 µm
Arora et al. (2015)	Rutile	50–75 µm	Rutile	106–125 µm
Sharma et al. [106]	Garnet	50–75 µm	Garnet	106–125 µm
Kumar et al. [107]	ZrSiO_4_	50–75 µm	Zircon	106–125 µm
Kumar et al. (2013)	ZrSiO_4_	3.75 µm	SiC	11.25 µm
Prabhu et al. (2017)	Nano clay	15–20 nm	CaSiO_3_	75–150 µm
Mizuuchi et al. [111]	Diamond	34.8 µm	Diamond	310 µm
Wang et al. [112]	SiC	0.2 µm	SiC	10 µm
Zhang et al. [113]	SiC	40 nm	SiC	15 µm
Avinash et al. [56]	SiC	38 µm	SiC	~250 µm
Malik et al. [114]	Al	45 µm	Cu	45 µm
Naik et al. [115]	CNT	Ø 15 nm & 6 µm length	Graphene	8 µm

## Data Availability

Not applicable.
